# The effect of musical interventions in improving short-term pain outcomes following total knee replacement: a meta-analysis and systematic review

**DOI:** 10.1186/s13018-020-01995-x

**Published:** 2020-10-09

**Authors:** Rongguo Yu, Youguang Zhuo, Eryou Feng, Wulian Wang, Wentao Lin, Feitai Lin, Zhanglai Li, Liqiong Lin, Lili Xiao, Haiyang Wang, Yuting Huang, Chunlin Wu, Yiyuan Zhang

**Affiliations:** Department of Orthopedics, Fuzhou second Hospital Affiliated to Xiamen University, Fuzhou, 350007 Fujian China

**Keywords:** Total knee replacement, TKR, TKA, Knee surgery, Music interventions, Music therapy, Pain, Meta-analysis, Systematic-review

## Abstract

**Background:**

A growing number of patients continue to receive total knee replacement (TKR) surgery. Nevertheless, such surgeries result in moderate to severe postoperative pain and difficulty in managing it. Musical interventions are regarded as a type of multimodal analgesia, achieving beneficial results in other clinical treatments. This study aims to evaluate the effect of musical interventions in improving short-term pain outcomes following TKR in order to determine a more reasonable and standard way of delivering musical intervention.

**Methods:**

A systematic search was conducted to identify available and relevant randomized controlled trials (RCTs) regarding musical interventions compared against non-musical interventions in patients treated with TKR in Embase, MEDLINE, Cochrane Library, Web of Science, CNKI, and Wanfang Med Online up to 8 January 2020. The authors independently assessed study eligibility and risk of bias and collected the outcomes of interest to analyze. The statistical analysis was conducted using the Review Manager (RevMan) version 5.30 software.

**Results:**

Eight RCTs comprised of 555 patients satisfied the inclusion criteria and were enrolled in the present study. The results showed no significant difference between the music and control groups in pain of the visual analog scale (VAS), during postoperative recovery room, back to the ward after surgery; anxiety degree of VAS; heart rate; respiratory rate; oxygen saturation; blood pressure, systolic blood pressure, and diastolic blood pressure. Nevertheless, significant differences were observed between the two groups in average increase in continuous passive motion (CPM) angles and LF/HF ratio (one kind index of heart rate variability).

**Conclusions:**

Musical interventions fail to demonstrate an obvious effect in improving short-term pain outcomes following TKR. A reasonable standardization of musical interventions, including musical type, outcome measures used, outcomes measured, duration, timing and headphones or players, may improve pain outcomes with certain advantages and should be further explored after TKR.

## Introduction

Total knee replacement has achieved good therapeutic effects in easing pain and improving functional outcomes for patients with rheumatoid arthritis or knee osteoarthritis [1, 2]. Approximately 100, 000 TKR operations are performed each year in the UK [3]. The number of TKR surgeries has been estimated to reach over 3.45 million by 2030 and increase by more than 670% by 2030 in the USA [4, 5]. Nevertheless, one study demonstrated that patients treated with TKR have been suffering from disproportionate pain, with 30% having moderate pain and 60% having severe pain [6]. Due to inadequate pain management following TKR, the procedure can lead to adverse outcomes such as severe anxiety, delayed convalescence, trouble with rest and sleep, reduced patient satisfaction, prolonged hospital stay, and heavier burden on healthcare [7–10]. It is a key focus of research, therefore, to improve patient satisfaction, optimize treatment, and reduce post-operation pain of TKR.

Chronic pain after TKR is caused by multiple factors such as psychological, biological, mechanical, surgical, and other factors [11–14]. Conventional studies have mainly focused on aspects of mechanical and biological using pharmacological interventions and physiotherapy [15, 16]. However, adverse drug reactions are important factors needing consideration like vomiting, nausea, dysarteriotony, urinary retention, and respiratory depression [17]. Non-pharmaceutical interventions have been regarded as simple, valuable, and low-cost supplementary methods in managing pain [18]. In the past two decades, a growing level of awareness regarding the hidden effects of music and other non-pharmacological strategies to improve postoperative results was present for interventions during the preoperative, perioperative, or postoperative periods [19, 20].

Musical therapies are natural interventions embodying multiple aspects like physical, emotional, psychological, spiritual recovery, and social. The proposed interventions have little side effects and high performance-cost ratio strategies that are easy to use and apply [21]. Music can stimulate the brain’s α waves, triggers relaxation and decreases muscular tension. It also stimulates the limbic system, leading to the release of endorphins, a type of neurotransmitter that causes a sense of well-being in humans [22]. Moreover, it can efficiently slow down the transmission of pain signals through the act of listening, alleviating the sense of pain [23], and reducing the dosage of paregoric post-surgery [24]. Listening to music stimulates the parasympathetic nervous system, restraining the action of the sympathetic nervous system, and reaching a target efficacy in easing anxiety [25, 26].

In practical clinic applications, nevertheless, incompatible consequences for musical interventions were present during the perioperative period, where various studies demonstrated pain relief [27–30] while others reported no significant changes [31]. Meanwhile, a number of studies carried out musical interventions solely within the operating room [30], while others applied them in post-anesthesia care units [32].

To the best of our knowledge, previous systematic reviews regarding psychological interventions in improving outcomes following TKR included musical therapy, hypnosis, guided imagery, and other different types of psychological interventions [33]. Due to its high heterogeneity, attaining a firm conclusion about the effectiveness of musical interventions in those treated with TKR is difficult. Furthermore, another meta-analysis and systematic review reported the effect of musical therapy on pain following orthopedic surgery, where musical interventions were found to relieve pain [34]. The study included a series of surgical operations when assessing the utility of listening to music after TKR, including the position of the knee, hip, shoulder, spine, and others. In similar circumstances, this study discussed improving outcomes for patients after TKR or THR [35]. The results are unconvincing because of the distinct prognosis and indications of two different surgical procedures.

Until now, no meta-analysis and systematic reviews have reported the effectiveness of musical interventions in patients who just received TKR. Due to the high incidence of pain following TKR, it may be of particular benefit to manage pain via musical interventions during the perioperative period. Therefore, a meta-analysis and systematic review was conducted to evaluate the effect of musical interventions in improving short-term pain outcomes after total knee replacement in order to ascertain a more reasonable and standard way of delivering musical interventions.

## Materials and methods

### Search strategies

This study was conformed to the Preferred Reporting Items for Systematic Reviews and Meta-Analyses (PRISMA) [36]. Comprehensive literature searches were conducted in Embase, MEDLINE, Cochrane Library, Web of Science, CNKI, and Wanfang Med Online databases for RCTs published from the earliest available records to 8 January 2020 using the following keywords and their combinations: total knee replacement, TKR, total knee arthroplasty, TKA, music, audio, music therapy, and music interventions. Both MeSH and Emtree headings were combined and were supplied with free text to enhance their sensitivity; however, they were also manually retrieved references from related research to ensure the inclusion of other studies. There were no language restrictions for searching.

### Inclusion and exclusion criteria

Studies were selected in the present research if they matched the following PICOS (population, intervention, comparator, outcome, study design) criteria: (I) Population, patients had received TKR; (II) Intervention, patients received musical interventions (music medicine or music therapy) intra-operatively, pre-operatively, or postoperatively; (III) Comparator, patients received an active treatment or control treatment (e.g., placebo, standard care, or no treatment); (IV) Outcomes such as assessment of pain severity during perioperative period, degree of anxiety, range of motion of the knee, physiological data including oxygen saturation, blood pressure, heart rate, heart rate variability, and respiratory rate; (V) Study design, RCTs.

The following conditions were considered exclusion criteria: revision knee arthroplasty; articles involving bilateral TKR; non-randomized trials; review articles; quasi-randomized trials; and articles with insufficient outcome data. The data were submitted to a third author for any divarication.

### Primary and secondary outcomes

This study only gathered statistical outcomes during the perioperative period. The primary outcomes included pain severity, while secondary outcomes included degree of anxiety, average increase in CPM angles, and physiological parameters including oxygen saturation, blood pressure, heart rate, LF/HF (heart rate variability) and respiratory rate.

### Data extraction

Final variables extracted included year of intervention, first author, country of origin, intervention time (perioperative, pre-operative, intra-operative, post-operative, or their combinations), type of music selection, music tracks, methodological characteristics, intervention details, participant characteristics, and measured outcomes. Two researchers independently extracted the data mentioned above. Discrepancies were discussed and resolved by additional authors. The corresponding authors of the primary studies were contacted to ensure that the integrated information was available. If there were multiple comparisons, only the interest data and information reported were extracted from the original studies.

### Quality assessment and risk of bias

Two authors independently evaluated the methodological quality according to Cochrane Handbook, version 5.1.0, for Systematic Reviews of Interventions (http://handbook.cochrane.org/). There were seven items to be included: (a) selection bias, random sequence generation; (b) selection bias, allocation concealment; (c) performance bias, blinding of the participants and personnel; (d) detection bias, blinding of outcome assessments; (e) reporting bias, selective reporting; (f) attrition bias, incomplete outcome data; (g) other biases. The entire methodological quality of each study in our review was measured as “yes” (low risk of bias), “unclear” (unclear risk of bias), or “no” (high risk of bias) and was used to obtain the risk of the bias graph and bias summary via Review Manager 5 (RevMan 5, version 5.30 Cochrane Collaboration, Oxford, UK). Any divided opinions were solved through team consensus.

### Statistical analysis

Review Manager 5 (RevMan 5) software (version 5.30, Cochrane Collaboration, Oxford, UK) was adopted for the statistical analysis in the present meta-analysis, where *P* value < 0.05 was considered to be statistically significant. Dichotomous outcomes were shown by risk difference (RD) and 95% confidence intervals (CIs). Continuous outcomes were shown by mean differences (MDs) and 95% CI, which was used for evaluation based on the *P* value and *I*^2^ value by the standard *χ*^*2*^ test. When the *I*^2^ < 50% or *χ*^*2*^ test > 0.1, manifesting significant heterogeneity, the fixed-effects model was adopted in the meta-analysis. Otherwise, the random-effects model was used. Sensitivity analysis was performed, if possible, to ascertain the origins of any heterogeneity.

## Results

### Study selection and characteristics of the selected studies

**Fig. 1:**
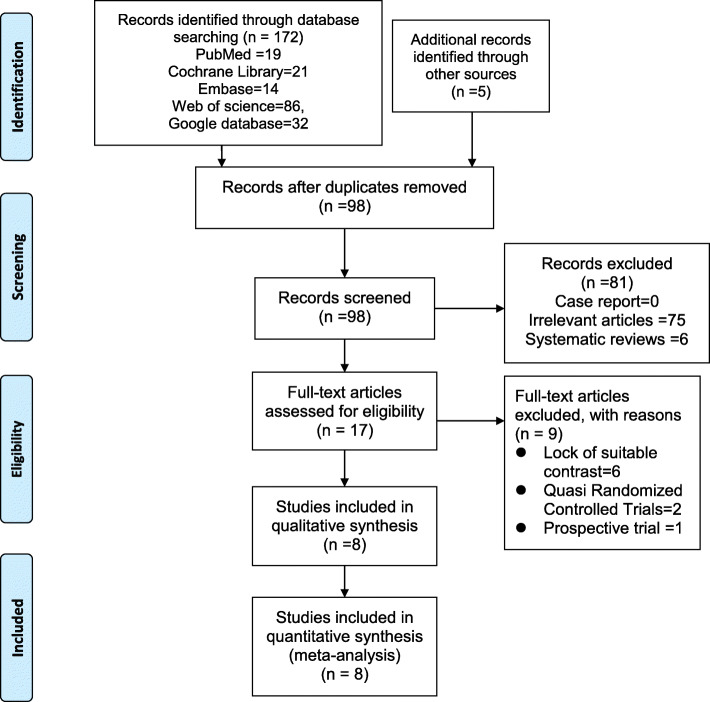
The PRISMA flow diagram detailing our literature search

In total, 172 studies were initially identified via electronic databases. Among the excluded articles, participators of three studies [37, 38] had both TKR and THA, and data in a particular study [39] had published in their anterior article [40]. Therefore, feasible data was unable to be acquired [41, 42]. After assessing the titles or abstracts, and after screening the full-texts of related studies, 8 RCTs satisfied the inclusion criteria [40, 43–49]. The present literature search was expounded by the PRISMA flow diagram (Fig. 1). All included articles were published between 2008 and 2019, and 555 participants were included in the analysis. The sample sizes of each study ranged from 32 to 117. The general characteristics of RCTs included in the meta-analysis are shown in Table 1, and the study clinic intervention protocol of RCTs included in the meta-analysis are put forward in Table 2.

**Table 1:** General characteristics of RCTs included in the meta-analysis

Studies	Year	Country	Type	Date of study	Sample size		Gender				Mean age		Timing of intervention	Primary results of statistics
					Music	Control	Music(F/M)		Control (F/M)		Music(M ± D)	Control(M ± D)		
Allred et al. [37]	2010	USA	RCT	In 2007	28	28	14	14	17	11	64.3 ±9.6	63.5 ± 9.6	Post-op	I. Pain: VAS and MPQ-SF II. Anxiety: VAS III. Physiologic data
Chen et al. [38]	2015	Taiwan	RCT	Not reported	30	30	20	10	25	5	69.86 ± 7.56^b^	69.86 ± 7.56^b^	Pre-op and post-op	I. Pain: VAS II. Physiological data III. Total amount of opioids used
Finlay et al. [39]	2016	UK	RCT	Not reported	72^a^	17	NS	NS	NS	NS	68.07 ± 8.03^b^	68.07 ± 8.03^b^	Pre-op and post-op	I. Pain: VRS/NRS II. Salivary cortisol concentrations III. Mood states: TMD
Hooks et al. [40]	2014	USA	RCT	September 2013 to November 2013	30	30	NS	NS	NS	NS	66.84 ± 66^b^	66.84 ± 66^b^	Post-op	I. Pain: NRS II. Physiological data III. Amount of opioids IV. LOS
Hsu et al. [41]	2015	Taiwan	RCT	November 2013 to April 2014	49	42	34	15	33	9	73.9 ± 7.5	71.33 ± 8.45	Post-op	I. Anxiety: VAS II. Physiological Parameters III. CPM angles 4.ROM
Leonard et al. [42]	2019	USA	RCT	Not reported	16	16	11	5	12	4	67.9(45–87)^c^	67.6(53–80)^c^	Post-op	I. Pain: NRS
Simcock et al. [43]	2008	USA	RCT	Junr 2006 to March 2007	60	57	37	23	33	24	31.3 ± 5.8	29.6 ± 6.1	Intra-op	I. Satisfaction scores II. Pain: VAS
You et al. [44]	2019	China	RCT	June 2018 to June 2018	25	25	NS	NS	NS	NS	65.2 ± 3.6^b^	65.2 ± 3.6^b^	Post-op	I. Physiological Parameters II. CPM angles III. ROM

*NS* not stated, *RCT* randomized controlled trial, *F/M* female/male, *M ± D* means ± standard deviation, *Post-op* = postoperative, *pre-op* preoperative, *Intra-op* intraoperative, *VAS* visual analog scale, *NRS* numerical rating scale, *MPQ-SF* McGill pain questionnaire short-form, *TMD* total mood disturbance, *LOS* length of stay, *CPM* continuous passive motion, *PPI* present pain intensity, *PRI* pain rating intensity, *ROM* range of motion

^a^ Containing four experimental groups

^b^The age of music group and control group

^c^Age range

**Table 2:** Clinic intervention protocol of RCTs included in the meta-analysis

Studies	Intervention details	Intervention and comparison	Type of music	Follow-up assessments	Intervention treatment	Control treatment
Allred et al.	Music via headphones	Music vs. quiet rest period	Easy listening	D1:T1 = 20 min before first PT session; T2 = just before PT; T3 = immediately after PT, T4 = 20 min after PT; T5 = 6 hours after intervention.	Listening to CD of easy listening music on headphones 20 min before first ambulation and for 20 min rest period after ambulation. Music had no lyrics, 60–80 beats/min.	20 min quiet rest period
Chen et al.	Music via broadcast speakers	Music vs. standard care	Soothing piano and violin	I. 10 min while the investigator prepared the study equipment at rest. II. In the surgical room in the morning. III. In the postoperative recovery area after the surgery. IV. Sending back to the ward One hour later.	Soothing piano and Chinese violin music played on a CD player through broadcast speakers. Played for 30 min in the preoperative ward, 30 min in the surgical room waiting area and 1 h in postoperative recovery.	Usual care
Finlay et al.	Music via headphones	Music vs. quiet bed rest	Varying degrees of harmonicity and rhythmicity	Pre-operative assessment at pre-admissions 2 weeks. All assessment measures were completed (D1–3) each day post-surgery. PCA usage was monitored pre-intervention in the immediate 24 h post-operatively (D0).	Four music-listening groups with four music types. Being visited daily and completing pre- and post-test at the same time each day, once per day for 3 days after surgery	Wearing noise canceling headphones with no input
Hooks et al.	Music via headphones	Music vs. quiet bed rest	Soft rock, jazz, Easy listening, R&B, Classical, Bluegrass, Country, Gospel, Pop, Nature sound	The patients were monitored in the morning between the times of 10:00 AM to 12:00 PM, early afternoon between 2:00 PM to 5:00 PM and evening between 7:30 PM to 9:30 PM on the first day after surgery.	The patients were asked not to alter the music player at any time. Before each session with the patients, I checked with the nurse and physical therapist to make sure the 30-min session would not interfere with the patient’s care plan.	wearing the ear buds for 30 min without music and the individual patient room door closed
Hsu et al.	Music via headphones	Music vs. standard care	Relaxing slow tempo, low tone, and soft melody	Receiving CPM rehabilitation twice daily (10 AM and 4 PM) on the first and second day following surgery	Listening to music from 10 min before receiving CPM until the end of the session (25 min in total) on the first and second day following surgery	Only to rest in bed 10 min before CPM.
Leonard et al	Music via music therapist	Music vs. standard care	Rock, Country, Traditional, Pop, Pop, Jazz, Bossa Nova	Baseline (1 min after flexion assessment), after each 2 min intervention period (two periods).	Music therapy during bicycling pedaling exercise postoperatively. Live music was played by a music therapist during PT supported pedaling exercise for 2 min, then pedaling alone with no music. Music included singing with paced guitar accompaniment and at a moderate/fast tempo.	Pedaling exercise with no music.
Simcock et al.	Music via headphones	Music vs. placebo	Patient’s choice what they like	Baseline, 3 h, 6 h, and 24 h after surgery procedure	Patient selection music during surgery by wearing headphones.	White noise control on headphones.
You et al.	Music via headphones	Music vs. quiet rest period	Soothing music	Preoperative , the first day and the second day after surgery during CPM	Starting CPM and listening to music until the end of the first 10 min during surgery by wearing headphones.	Usual care

*D* postoperative day, *PT* physical therapy, *CPM* continuous passive motion

### Quality assessment and risk of bias

**Fig. 2:**
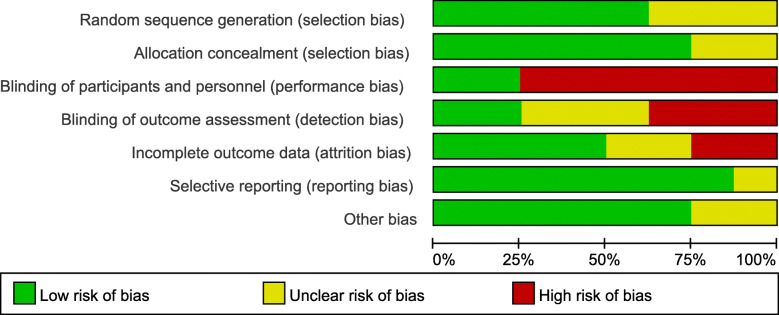
The risk of bias summary of the included studies. (+ represents yes; – represents no; ? represents not clear)

**Fig. 3:**
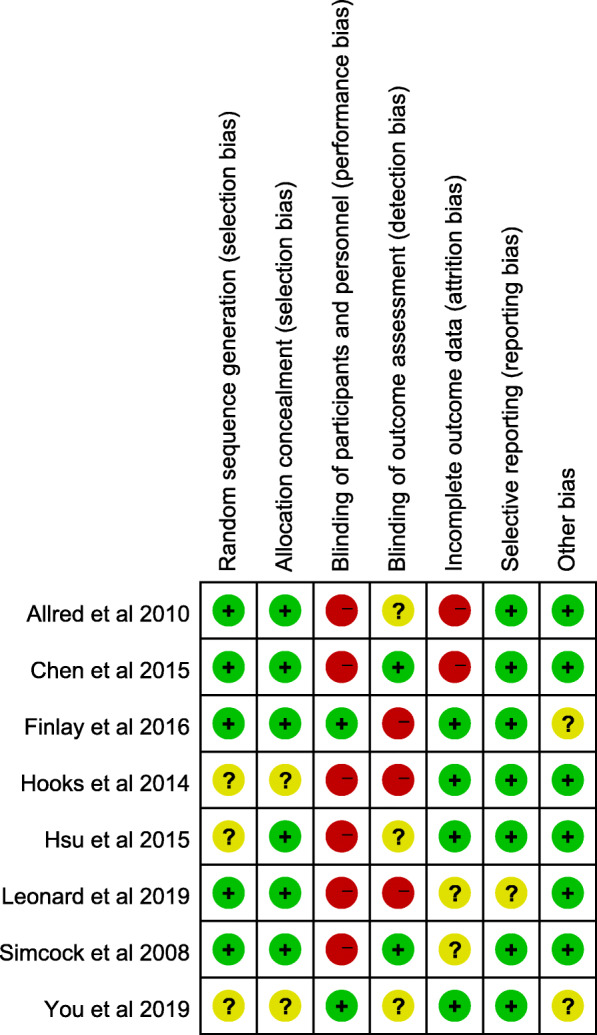
The risk of bias graph of the included studies.

The present study was evaluated according to the Cochrane Handbook for Systematic Reviews of Interventions in terms of the methodological quality of all included RCTs. Five RCTs [43–45, 47, 48] mentioned the sequence generation (randomization scheme performed) fairly well, and six trials mentioned allocation concealment [40, 43–45, 47, 48]. In the remaining articles, this information was indistinct or absent [46, 49]. Blinding of personnel and participants was mentioned in two trials [46, 49], but was not performed in six trials [40, 43, 44, 46–48]. In regard to outcome assessors, two trials were blinded [44, 48], though three trials were unclear [40, 43, 49]. Blinding was not performed in the other three studies [45–47]. Furthermore, no other apparent bias was found in each included study. All included RCTs were considered to be low risk for attrition bias and furnished complete data. The detailed risks of bias for the eligible studies are shown in Figs. 2 and 3.

### Primary outcomes

#### Meta-analysis of pain severity

**Fig. 4:**
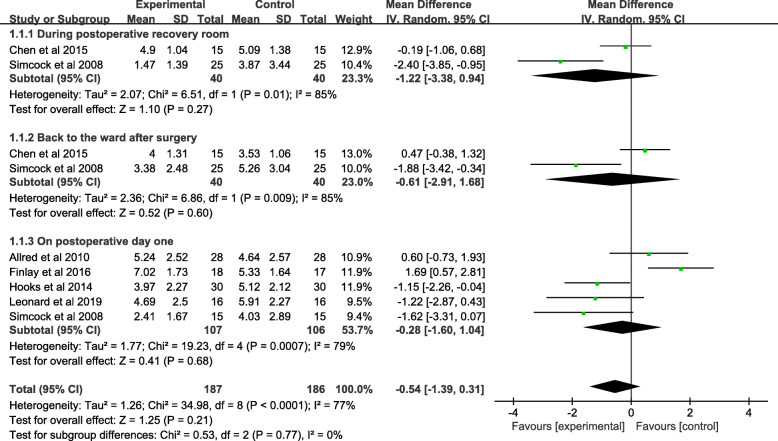
A forest plot diagram showing the pain severity

A total of 373 patients reported pain severity in 6 studies. Two studies assessing 80 knees involved pain of VAS in the postoperative recovery room. Moreover, no significant difference was evident between the music and control groups (MD = − 1.22; 95% CI; − 3.38 to 0.94; *P* = 0.27). Only 2 studies (80 patients) reported pain of VAS back to the ward after surgery, where no significant difference was found between the music and control groups (MD = − 0.61; 95% CI; − 2.91 to 1.68; *P* = 0.60). Additionally, 5 studies (213 patients) stated related pain in the VAS score on the postoperative day (POD) 1. The pooled data showed no significant difference between the two groups (MD = − 0.28; 95% CI; − 1.60 to 1.04; *P* = 0.68). In terms of the high heterogeneity of the three subgroups following, during postoperative recovery (*χ*^*2*^ = 6.51; df = 1; *P* = 0.01; *I*^*2*^ = 85%); back to the ward (*χ*^*2*^ = 6.86; df = 1; *P* = 0.009; *I*^*2*^ = 85%), POD1 (*χ*^*2*^ = 19.23; df = 2; *P* = 0.0007; *I*^*2*^ = 79%), a random-effects model was used (Fig. 4 and Table 3).

**Table 3:** Clinical results of meta-analysis

Clinical results	Studies	Number of participants			Incidence				
		Total	Music	Control	*P*	MD	95% CI	Heterogeneity P (*I*^2^)	Model
Pain severity									
During PRR	2	80	40	40	0.27	− 1.22	− 3.38 to 0.94	0.01(85%)	Random
Back to the ward after surgery	2	80	40	40	0.60	− 0.61	− 2.91 to 1.68	0.009(85%)	Random
On POD 1	5	213	107	106	0.68	− 0.28	− 1.60 to 1.04	0.0007(79%)	Random
Anxiety degree									
Before PT on POD 1	2	147	77	70	0.87	− 0.18	− 2.35 to 1.99	0.004 (88%)	Random
After PT on POD 1	2	147	77	70	0.20	− 1.49	− 3.78 to 0.79	0.001(91%)	Random
Average increase in CPM angles									
On POD 1	2	141	74	67	0.0008	8.90	3.72 to 14.08	0.01(84%)	Random
On POD 2	2	141	74	67	< 0.00001	4.24	2.15 to 6.32	0.13(56%)	Random
Heart rate									
Before PT on POD 1	2	147	77	70	0.54	1.23	− 2.66 to 5.13	0.49(0%)	Fixed
After PT on POD 1	3	207	107	100	0.97	0.31	− 3.53 to 3.65	0.31(16%)	Fixed
Blood pressure									
Systolic blood pressure	2	90	45	45	0.52	− 2.76	− 11.10 to 5.58	0.46(0%)	Fixed
Diastolic blood pressure	2	90	45	45	0.38	− 1.80	− 5.78 to 2.19	0.83(0%)	Random
Respiratory rate	2	116	58	58	0.56	0.14	− 0.33 to 0.61	0.93(0%)	Fixed
Oxygen saturation	2	116	58	58	0.22	− 0.51	− 1.32 to 0.31	0.50(0%)	Fixed
LF/HF ratio									
Before PT on POD 1	2	141	74	67	< 0.00001	− 1.00	− 1.23 to − 0.78	0.96(0%)	
After PT on POD 1	2	141	74	67	< 0.00001	− 1.40	− 1.50 to − 1.30	0.98(0%)	Fixed
Before PT on POD 2	2	141	74	67	< 0.00001	− 0.90	− 0.98 to − 0.82	0.95(0%)	Fixed
After PT on POD 2	2	141	74	67	< 0.00001	− 1.60	− 1.71 to − 1.49	1.00(0%)	Fixed

*PRR* postoperative recovery room, *MD* mean difference, *CI* confidence interval, *FPM* fast-paced music, *POD* postoperative day, *CPM* continuous passive motion, *LF/HF* one kind index of heart rate variability

### Secondary outcomes

#### Meta-analysis of anxiety degree

**Fig. 5:**
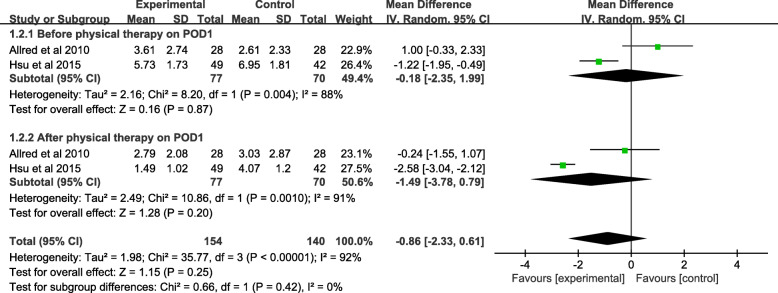
A forest plot diagram showing the anxiety degree

Only 2 studies (147 patients) reported the anxiety degree of VAS scores before PT on POD1. No significant difference was found between the two groups (MD = − 0.18; 95% CI; − 2.35 to 1.99; *P* = 0.87). The anxiety degree of VAS scores after PT on POD1 was reported in tow of the included studies, and a total of 147 patients were involved in the meta-analysis. The pooled data showed no significant difference between the music and control groups (MD = − 1.49; 95% CI; − 3.78 to 0.79; *P* = 0.20). Finding the high heterogeneity in before PT (*χ*^*2*^ = 8.20; df = 1; *P* = 0.004; *I*^*2*^ = 88%) and after PT (*χ*^*2*^ = 10.86; df = 1; *P* = 0.001; *I*^*2*^ = 91%), a random-effects model was adopted (Fig. 5 and Table 3).

#### Meta-analysis of the average increase in CPM angles

**Fig. 6:**
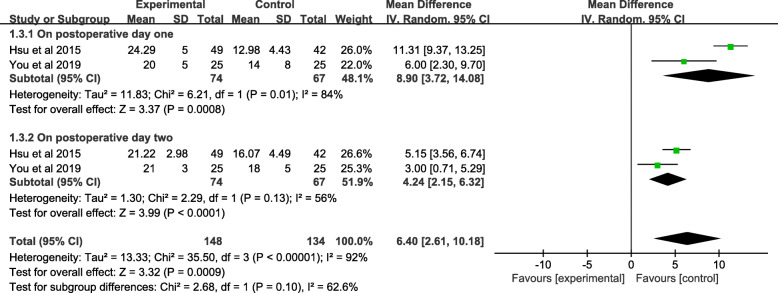
A forest plot diagram showing the average increase in CPM angles

A total of 141 patients reported an average increase in CPM angles in tow studies on POD1. There were significant differences between the music and control groups (MD = 8.90; 95% CI; 3.72 to 14.08; *P* = 0.0008). Two studies assessing 141 knees involved an average increase in CPM angles POD2, and a significant difference was observed between the included studies (MD = 4.24, 95% CI 215 to 6.32, *P* < 0.00001). In view of the high heterogeneity in the tow subgroups, POD1 (*χ2* = 6.21; df = 1; *P*= 0.01; *I*^2^ = 84%) and POD2 (*χ2* = 3.99; df = 1; *P* = 0.13; *I*^2^ = 56%), a random-effects model was utilized (Fig. 6 and Table 3).

#### Meta-analysis of heart rate

**Fig. 7:**
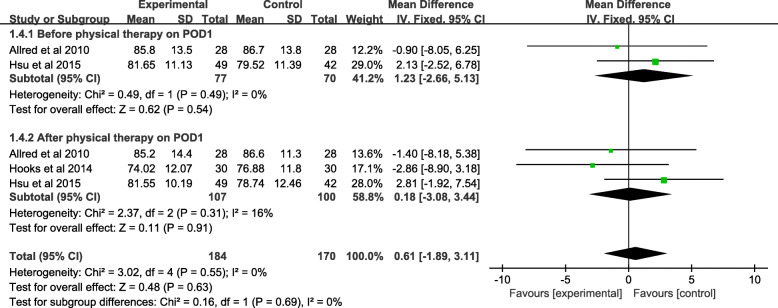
A forest plot diagram showing the heart rate

Heart rate before PT on postoperative day (POD) 1 was reported in two studies, where a total of 147 patients was involved in the present research. The pooled data showed no significant difference between the two methods of interventions (MD = − 1.23, 95% CI − 2.66 to 5.13, *P* = 0.54). Three trails (207 patients) reported the heart rate after PT on postoperative day (POD) 1. The pooled data showed no significant difference (MD = − 0.06, 95% CI − 3.53 to 3.65, *P* = 0.97) between the two methods of interventions. Due to the important heterogeneity in heart rate before PT on POD 1 (*χ*^*2*^ = 0.49; df = 1; *P* = 0.49; *I*^*2*^ = 0%) and heart rate after PT on POD 1 (*χ*^*2*^ = 2.37; df = 2; *P* = 0.31; *I*^*2*^ = 16%), a fixed-effects model was applied (Fig. 7 and Table 3).

#### Meta-analysis of blood pressure

**Fig. 8:**
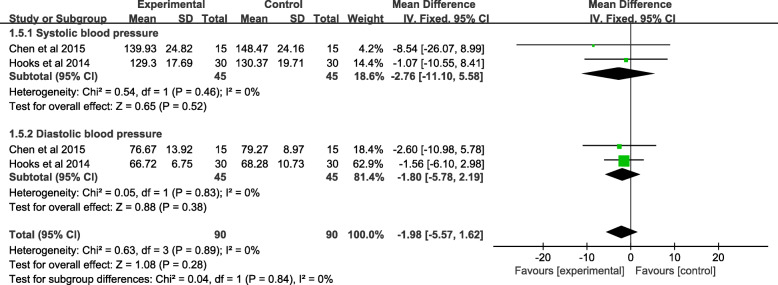
A forest plot diagram showing the blood pressure

Two studies comprised of 90 patients reported systolic blood pressure, showing no statistical significance between the music and control groups (MD = − 2.76, 95% CI − 11.10 to 5.58, *P* = 0.52). The data of diastolic blood pressure was reported by two trails (147 patients). No significant difference was found between the two groups (MD = − 1.80, 95% CI − 5.78 to 2.19, *P* = 0.38). A random-effects model was used due to significant heterogeneity, systolic blood pressure (*χ*^*2*^ = 0.54; df = 1; *P* = 0.46; *I*^*2*^ = 0%), and diastolic blood pressure (*χ*^*2*^ = 0.05; df = 2; *P* = 1; *I*^*2*^ = 0%), which was found in the data of blood pressure (Fig. 8 and Table 3).

#### Meta-analysis of respiratory rate

**Fig. 9:**
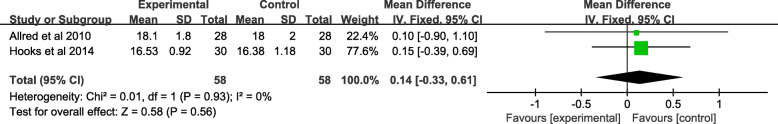
A forest plot diagram showing the respiratory rate

Only 2 studies (116 patients) reported the respiratory rate after PT on POD1. No significant difference was found between the two groups (MD = 0.14; 95% CI − 0.33 to 0.61; *P* = 0.56). A fixed-effects model was used as significant heterogeneity was found in the data of respiratory rate (*χ*^*2*^ = 0.01; df = 1; *P* = 0.93; *I*^*2*^ = 0%) (Fig. 9 and Table 3).

### Meta-analysis of oxygen saturation

**Fig. 10:**
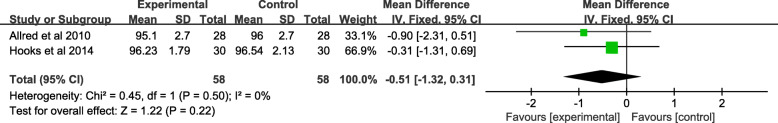
A forest plot diagram showing the oxygen saturation

Oxygen saturation was reported in two studies, where a total of 116 knees were involved in the meta-analysis. There was no significant difference between the music and control groups (MD = − 0.51; 95% CI − 1.32 to 0.31; *P* = 0.22). In view of low heterogeneity (*χ*^*2*^ = 0.45; df = 1; *P* = 0.50; *I*^*2*^ = 0%), a fixed-effects model was applied (Fig 10 and Table 3).

#### Meta-analysis of LF/HF ratio

**Fig. 11:**
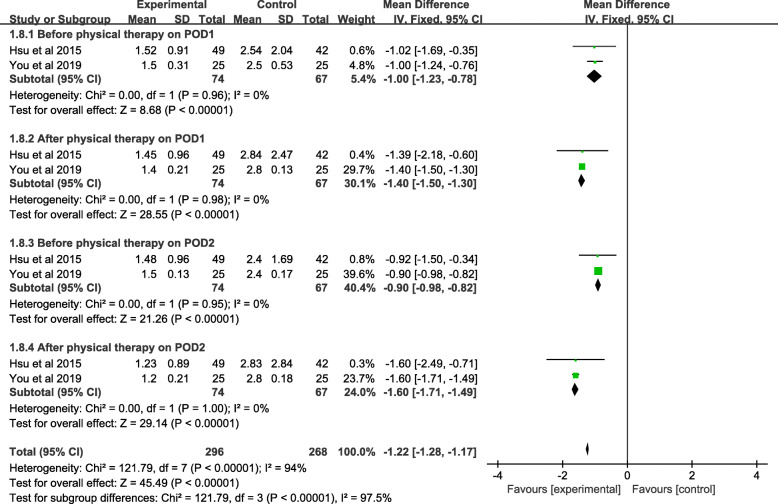
A forest plot diagram showing the LF/HF ratio

Two studies (141 patients) reported the LF/HF ratio before PT on POD 1, and a significant difference was shown between the included studies (MD = − 1.00, 95% CI − 1.23 to − 0.78, *P* < 0.001). Two studies with 141 patients reported the LF/HF after PT on POD1, demonstrating a statistical significance between the music and control groups (MD = − 1.40, 95% CI − 1.50 to − 0.30, *P* < 0.001). Data pertaining to LF/HF before PT on POD 2 was reported by two trials of 141 patients. A significant difference was found between the two groups (MD = − 0.90, 95% CI − 0.98 to − 0.82, *P* < 0.001). Data regarding the LF/HF after PT on POD 2 was reported in two of the included studies, where a total of 141 patients were involved in the meta-analysis. The pooled data showed a significant difference between the music and control groups (MD = − 1.60; 95% CI − 1.71 to − 1.49; *P* < 0.001). Low heterogeneity of the four subgroups was observed, where POD1: before PT(*χ*^*2*^ = 0.00; df = 1; *P* = 0.98; *I*^*2*^ = 0%), POD1: after PT(*χ*^*2*^ = 0.00; df = 1; *P* = 0.98; *I*^*2*^ = 0%), POD2: after PT(*χ*^*2*^ = 0.00; df = 1; *P* = 0.95; *I*^*2*^ = 0%), POD2: before PT(*χ*^*2*^ = 0.00; df = 1; *P* = 1.00; *I*^*2*^ = 0%), a fixed-effects model was used (Fig. 11 and Table 3).

### Subgroup analysis of others

A subgroup analyses were conducted to assess the effects of music via headphones as well as fast-paced music between the two groups (Table 4). The outcomes indicated that patients in the music groups had no significant difference in respiratory rate, heart rate, pain severity, oxygen saturation, and blood pressure compared to the control groups with or without music via headphones and fast-paced music.

**Table 4:** Results subgroup analysis of others

Clinical results	Studies	Number of participants			Incidence				
		Total	Music	Control	*P*	MD	95% CI	Heterogeneity P (*I*^2^)	Model
Respiratory rate									
Headphones	2	116	58	58	0.76	0.06	− 0.35 to 0.48	0.80(0%)	Fixed
Non-headphones	1	30	15	15	0.22	0.14	− 0.88 to 1.17	NS	NS
Including FPM	1	60	30	30	0.58	0.15	− 0.39 to 0.69	NS	NS
Not including FPM	2	86	43	43	0.98	− 0.01	− 0.52 to 0.51	0.62(0%)	Fixed
Heart rate									
Headphones	3	207	107	100	0.74	0.34	− 1.68 to 2.37	0.79(0%)	Fixed
Non-headphones	1	30	15	15	1.00	− 0.02	− 6.36 to 6.33	NS	NS
Including FPM	1	60	30	30	0.35	− 2.86	− 8.90 to 3.18	NS	NS
Not including FPM	3	187	92	95	0.52	0.67	− 1.37 to 2.71	0.95(0%)	Fixed
Pain severity									
Headphones	4	182	91	91	0.28	− 0.74	− 2.09 to 0.60	< 0.00001(83%)	Random
Non-headphones	2	62	31	31	0.96	− 0.02	− 0.59 to 0.56	0.18(42%)	Fixed
Including FPM	4	157	79	78	0.12	− 1.05	− 2.37 to 0.28	< 0.00001(82%)	Random
Not including FPM	2	86	43	43	0.42	0.23	− 0.33 to 0.78	0.48(0%)	Fixed
Oxygen saturation									
Including FPM	1	60	30	30	0.54	− 0.31	− 1.31 to 0.69	NS	NS
Not including FPM	1	56	28	28	0.21	− 0.90	− 0.90 to 0.51	NS	NS
Blood pressure									
Headphones	1	60	30	30	0.48	− 1.47	− 5.56 to 2.26	NS	NS
Non-headphones	1	30	15	15	0.34	− 3.71	11.27 to 3.86	NS	NS

*MD* mean difference, *CI* confidence interval, *FPM* fast-paced music, *NS* not state

## Discussion

Recently, an increasing number of patients received TKR [50]; however, pain management remains unsatisfactory following surgery [51]. Epidural analgesia, patient-controlled analgesia (PCA), and other medications serve as conventional methods in controlling pain [52–54], which may be associated with side effects. Multimodal analgesia implements at least two separate modalities of analgesia [55], and non-pharmacologic approaches are one aspect of multimodal therapy [56]. Music may be regarded to be very effective in terms of non-pharmacological pain management strategies [57]; however, its efficacy is known in relation to patients treated with TKR. This study is the first systematic review and meta-analysis evaluating the efficiency of musical interventions on patients treated with TKR, which attempted to determine a more reasonable and standard implementation of musical interventions.

### Pain severity

In the present meta-analysis, no significant difference was observed for VAS scores during the postoperative recovery room, back to the ward after surgery, and postoperative day 1 between the music and control groups. The corresponding result is at variance with previous outcomes. First, researchers have previously shown the positive effects of musical interventions in different aspects of treatment such as cesarean section, intestinal, gynecologic, and nasal care [58–61]. Orthopedic surgeries are frequently associated with different outcomes in other types of surgery. Due to copious amounts of bone destruction and soft tissue injury, orthopedic surgery is generally associated with insufferable post-surgical pain [62]. Second, one study found that musical interventions performed immediately after TKR obviously alleviated pain over time [43] and mentioned that such interventions resulted in reduced opioid dosages and incidence of adverse reactions. The best efforts were made in collecting data long after having TKR as this may make a difference to a certain degree but failed due to the limited number of RCTs. Third, musical interventions could, in theory, shift one’s attention [63, 64], make people relax [65], and increase one’s sense of well-being [22]. We consider the main reason why it is different from us is the increased pain due to functional exercise in order to attain better knee flexion angles, which is a key result indicator of TKR surgery [66]. This study did not exclude the possibility of being influenced by subjective factors as the VAS score is a subjective scale. In addition, the heterogeneity of musical interventions should be noted as it also leads to divergence.

### Anxiety degree

Previous studies have reported postoperative decreased anxiety with intraoperative music use in orthopedic surgery [67]. Due to limited studies, the present meta-analysis only collected data regarding before and after physical therapy with postoperative music use on POD 1. The results of the combined analysis showed that the music and control groups had no significant difference in anxiety degree after TKR. This outcome is similar to that of Allred and his colleagues [43], though another study found positive outcomes [40], which may be explained by the timing of the musical interventions. In Allred’s study, they rendered their music before and after initial physical therapy. However, Hsu and his colleagues provided music in three moments: before, during and after physical therapy. Therefore, we assume whether increasing the frequency of music may be associated with a positive efficiency. A similar assumption was supported in a previous systematic review [34], but varying surgical procedures may result in unreliable evidence. As correlation studies are limited in TKR surgery, this study failed to obtain an accurate answer. In addition, it is worth noting that a sense of stress can excite or stimulate a patient’s sympathetic nervous system, facilitating anxiety [68].

### The average increase in CPM angles

The average increase in CPM angles was the secondary outcome assessed in this meta-analysis. Accordingly, significant differences were noted in the first and second days following surgery between the two groups. Many researchers posited that postoperative knee flexion angle is one of the main prognostic indexes of TKR surgery [69–72], and treatment of CPM was current clinical common practice [73, 74]. To this effect, CPM was reported to increase the voluntary knee joint ROM angle by an average of 4.3° in a short period [70]. As CPM treatment stretches surgical wounds, patients suffered from severe pain during this process. Therefore, certain patients were unsuccessful in complying with daily CPM rehabilitation. As the feeling of fear caused their muscles to tighten, the effectiveness of CPM was impacted. Patients who are relaxed during rehabilitation would improve the effectiveness of the treatment [75]. Music is known to be an art that promotes relaxation, which theoretically make sense. However, more RCTs are needed to confirm the degree of relaxation.

### Physiologic parameters

Thus far, adopting musical treatment in clinical practice remains ambiguous. In order to generate further discussion, physiological parameters serve as an important component in accessing interventions. This study collected multiple physiologic parameters including oxygen saturation, blood pressure, heart rate, LF/HF ratio, and respiratory rate, though only LF/HF showed statistical significance. Similar results were reported by another study, where they found a lack of statistical difference among diastolic blood pressure, systolic blood pressure, and heart rate in participants in a rest group (*n* = 20) as well as a musical intervention group (*n* = 50) [76]. LF was used to describe the sympathetic activity and was of positive relevance with anxiety, however, HF indicated parasympathetic activity [77]. Furthermore, the LF/HF ratio evaluated the quantity of sympathetic balance to some extent [78], as well as a type of evaluation index concerning heart rate variability [79]. Low LF/HF ratio values indicate strong parasympathetic activity, whereas high values show strong sympathetic activity. Studies in other fields of surgery demonstrated similar ratios as this study [39, 78]. Other holistic studies reported that musical interventions decreased physiological stress parameters and perioperative stress hormones. These results were not supported by powerful evidence [61, 80]. Nevertheless, another article considered only sparse evidence regarding the effectiveness of music in blood pressure, heart rate, and respiratory rate during the preoperative, postoperative, or intraoperative periods [20]. Surgery may influence the cardiovascular system by stimulating the sympathetic nervous system like in elevated blood pressure, heart rate, and oxygen saturation [81]. Additional research in this regard could provide the relevant data in these fields.

### Subgroup analysis of others

During data processing, various modes of musical interventions were identified, which led to high heterogeneity in the systematic review. Consequently, subgroup analyses were performed to assess the effects of music via headphones and fast-paced music between the two groups in order to determine a more reasonable and standard way of musical intervention. Due to the limited number of studies, this study failed to obtain a definite conclusion. More than one study demonstrated large discrepancies in the studied intervention type, outcome measures used, outcomes measured, duration, timing of intervention participants, and choices of music [82, 83]. Participants with headphones in the control group reported that headphones relieved anxiety by shielding background noise in the postoperative recovery room [84]. In addition, headphones promoted privacy, avoiding external noise, reducing noise-related mental fatigue, and more [22, 85, 86]. The music preferences of patients were a key factor when opting for musical intervention. This involved a patient-centered practice by allowing patients to choose the type of rhythm, which may achieve better results [87]. Interestingly, a previous study found that melodies were preferred to rhythms as melodies could alter the activity level of the adrenergic system [88]. Due to this study’s small sample size, the results should be interpreted with caution as additional related studies are undertaken.

### Limitations

This research is the first meta-analysis and systematic review that appraises the effectiveness of musical interventions for TKR patients. However, various limitations were present in this study. Due to heterogeneity in the intensity, duration, type of music, and type of the interventions, few probabilities in acquiring abundant data exist in this study, which increased the difficulty in generalizing the findings for this population. In addition, high-quality studies may have been overlooked. Moreover, the conclusions may have been affected as some of the included studies had noticeable methodological shortcomings. Additionally, the studies in the present meta-analysis had unclear or high risk-of-bias ratings, which may cause the conclusion to lack persuasiveness. However, despite these limitations, a comprehensive review was given to estimate the effect of musical interventions in improving short-term pain outcomes following TKR, while determining meaningful methodological instructions for future studies.

## Conclusion

Musical interventions fail to demonstrate an obvious effect in improving short-term pain outcomes following TKR. This review also repeatedly emphasizes the need for additional evidence in exploring the standardization of musical interventions (including musical type, outcome measures used, outcomes measured, duration, timing, and headphones or players) in improving pain outcomes after TKR, paving the way for future studies. The number and quality of the included studies were limited; consequently, studies with properly randomized techniques and larger sample sizes are anticipated.

## Data Availability

We state that all data generated during the present study are included in this article.

## Abbreviations

CI: Confidence interval
CPM: Continuous passive motion
LOS: Length of stay
LF/HF: Low-frequency/high-frequency ratio
MD: Mean difference
NS: Not stated
Intra-op: Intraoperative
POD: Postoperative day
Pre-op: Preoperative
PRISMA: Preferred Reporting Items for Systematic Reviews and Meta-Analyses
PT: Physical therapy
Post-op: Postoperative
RCTs: Randomized controlled trials
RD: Risk difference
RevMan: Review Manager
ROM: Range of motion
TKA: Total knee arthroplasty
TKR: Total knee replacement
VAS: Visual analog scale
